# Effects of Decursin and *Angelica gigas* Nakai Root Extract on Hair Growth in Mouse Dorsal Skin via Regulating Inflammatory Cytokines

**DOI:** 10.3390/molecules25163697

**Published:** 2020-08-13

**Authors:** Tae-Kyeong Lee, Bora Kim, Dae Won Kim, Ji Hyeon Ahn, Hyejin Sim, Jae-Chul Lee, Go Eun Yang, Young Her, Joon Ha Park, Hyun Sook Kim, Tae Heung Sim, Hyun Sam Lee, Moo-Ho Won

**Affiliations:** 1 Department of Biomedical Science and Research Institute for Bioscience and Biotechnology, Hallym University, Chuncheon, Gangwon 24252, Korea; tk-lee@hallym.ac.kr (T.-K.L.); jh-ahn@hallym.ac.kr (J.H.A.); 2 Department of Neurobiology, School of Medicine, Kangwon National University, Chuncheon, Gangwon 24341, Korea; bora9417@kangwon.ac.kr (B.K.); janny20@kangwon.ac.kr (H.S.); anajclee@kangwon.ac.kr (J.-C.L.); 3 Department of Biochemistry and Molecular Biology, and Research Institute of Oral Sciences, College of Dentistry, Gangnung-Wonju National University, Gangneung, Gangwon 25457, Korea; kimdw@gwnu.ac.kr; 4 Department of Radiology, Kangwon National University Hospital, Kangwon National University School of Medicine, Chuncheon, Gangwon 24289, Korea; yangke@kangwon.ac.kr; 5 Department of Dermatology, Kangwon National University Hospital, Kangwon National University School of Medicine, Chuncheon, Gangwon 24289, Korea; youngderma@knuh.or.kr; 6 Department of Anatomy, College of Korean Medicine, Dongguk University, Gyeongju, Gyeongbuk 38066, Korea; jh-park@dongguk.ac.kr; 7 Leefarm Co., Ltd., Hongcheon, Gangwon 25117, Korea; K18860@naver.com (H.S.K.); 119ato@naver.com (T.H.S.)

**Keywords:** *Angelica gigas* Nakai, decursin, hair growth facilitator, hair loss, inflammation

## Abstract

This current study investigates the facilitative effects and mechanisms of decursin, a major component of *Angelica gigas* Nakai (AGN), and AGN root extract on hair growth in mice. We perform high-performance liquid chromatography on AGN extract to show it contains 7.3% decursin. Hairs in mouse dorsal skin are shaved distilled in water, 0.15% decursin, and 2% AGN root extract (0.15% decursin in the diluted extract) and topically applied twice a day for 17 days. Hematoxylin and eosin staining are done to examine the morphological changes in the hair follicles. To compare the effects of decursin and AGN extract on inflammatory cytokines in the dorsal skin, Western blot analysis and immunohistochemistry for tumor necrosis factor α (TNF-α) and interleukin (IL)-1β as pro-inflammatory cytokines, and IL-4 and IL-13 as anti-inflammatory cytokines are conducted. The results show that the application of decursin and AGN extract confer effects on hair growth. Hair growth is significantly facilitated from seven days after the treatments compared to that in the control group, and completely grown hair was found 17 days after the treatments. The protein levels and immunoreactivity of TNF-α and IL-1β in this case are significantly decreased, whereas the IL-4 and IL-13 levels and immunoreactivity are significantly increased compared to those in the control group. Additionally, high-mobility group box 1, an inflammatory mediator, is elevated by the topical application of decursin and AGN extract. Taken together, the treatment of mouse dorsal skin with AGE root extract containing decursin promotes hair growth by regulating pro- and/or anti-inflammatory cytokines. We, therefore, suggest that AGN root extract as well as decursin can be utilized as materials for developing hair growth-facilitating treatments.

## 1. Introduction

Hair provides protection, regulates body temperature, facilitates evaporation of perspiration, and functions as a sensory organ [1]. Hair generally has been regarded as the main representative feature of each individual [2]. Hair loss not only affects the functions of hair but also negatively affects psychological aspects, such as causing a decline in self-esteem [2,3]. It has been well acknowledged that hair loss is caused by diverse reasons including family history, hormonal imbalances, extreme stress, and specific medications, including retinoids, chemotherapy, and anti-thyroid agents. Thus, different treatments and care are recommended [2,4].

Inflammatory responses are fundamentally triggered by activated immune cells according to the invasion of antigens [5]. To contrast, fine regulations in inflammatory responses are accomplished through the antagonism between pro-inflammatory cytokines, such as tumor necrosis factor α (TNF- α) and interleukin (IL)-1β, and anti-inflammatory cytokines, such as IL-4 and IL-13 [6,7,8]. Some studies have demonstrated the relationship between inflammatory responses and hair loss [9,10]. Particularly, the inflammatory response in hair follicles critically affects hair loss [11].

*Angelica gigas* Nakai (AGN) belongs to the Umbelliferae family and has been traditionally used in oriental remedies due to its many advantageous attributes, such as antiepileptic, hepatoprotective, anti-cancer, anti-inflammatory, and anti-amnesic activities [12,13,14,15,16,17]. Concerning the case of hair growth using AGN, the extract of the roots of AGN is frequently included as a hair growth-facilitating component in combined herbal extracts [18,19,20,21].

To the best of our knowledge, however, the promotive effect of decursin and AGN root extract on hair growth and their mechanisms have not been fully investigated yet. A recent study reported that decursin is a major component of root-exerted anti-inflammatory effects [22]. Therefore, the main objective of the current study is to investigate the effects of decursin and AGN root extract on hair growth in mouse dorsal skin and expressions of pro- and anti-inflammatory cytokines in the dorsal skin. Additionally, we examine alterations in high-mobility group box 1 (HMGB1), an inflammatory mediator.

## 2. Materials and Methods

### 2.1. Preparation of Decursin and AGN Root Extract

Decursin is a coumarin derivative compound and considered a major component of AGN root extract. We purchased decursin from ChemFaces (Cat. No. CFN98509; Wuhan, Hubei, China). AGN was provided from KGC Yebon Co. Ltd. (Chungju, Republic of Korea) which was manufactured with a standardized extracting process. AGN was cultivated in Chuncheon (Republic of Korea). The roots of AGN were harvested and rinsed with pure water followed by dehydration. Next, the dried AGN roots were crushed into a fine powder using a grinder (IKA M20, IKA, Staufen, Germany). Ethanol solution (98%, *v/v*) was used as an extract solvent. The volume of the ethanol solution was five times the volume of the powder, and the process of the extraction took 4 h in 55 ± 5 °C for the extraction solvent. Thereafter, the AGN root extract was filtered via a Whatman No. 1 (Whatman Ltd., Maidstone, Kent, UK) and concentrated at 45 ± 5 °C in a vacuum evaporator until the final weight was 10% versus the original weight. Finally, the AGN root extract was completely dried and in powder form.

### 2.2. Qualitative Analysis of AGN Extract

To qualitatively analyze the AGN extract, high-performance liquid chromatography (HPLC) was carried out in accordance with a previously described method with some modifications [23]. Briefly, AGN extract (test solution) and decursin (standard solution) were precisely weighed (1 g and 0.01 g respectively) and dissolved in 50 mL methanol (50% *v/v* in distilled water). Using a stainless column (inner diameter, 4.6 mm; length, 250 mm), which was filled with octadecylsilyl silica gel (diameter, 5 μm), 10 μL of AGN extract and decursin were respectively chromatographed. The mobile phases were set as A (acetonitrile) and B (distilled water) under gradual concentrations (0 min, 20% A; 3 min, 20% A; 8 min 30% A; 18 min, 30% A; 19 min, 50% A; 40 min, 50% A; 41 min, 90% A; 50 min, 90% A). Using a UV-spectrophotometer (wavelength, 330 nm) the ingredients of the AGN extract were detected with 1.0 mL/min of flow rate.

### 2.3. Experimental Animals

Male C57/BL6 mice (total *n* = 51; age, 6–7 weeks; body weight, 25 ± 2 g) were acquired from the Experimental Animal Center of Kangwon National University (Chuncheon, Republic of Korea). They were cared for in conventional conditions under an optimal indoor temperature (25 ± 0.5 °C) and relative humidity (60 ± 5%). The conventional 12:12 light–dark cycle (light on at 6:00 a.m.) was kept. Sufficient feed and water were supplied to the mice.

The experimental protocol of this study was approved by the Institutional Animal Care and Use Committee (IACUC) in Kangwon National University (approval No. KW-190128-1). The protocol included the handling and management of the experimental animals, which was exhaustively maintained at the Current International Laws and Policies in the “Guide for the Care and Use of Laboratory Animals” (The National Academies Press, 8th Ed., 2011).

### 2.4. Experimental Groups and Treatments of Decursin and AGN Extract

Mice were randomly classified into three groups (n = 17 in each group): (1) the control group, which was treated with distilled water (DW) as a vehicle; (2) the decursin group, which was treated with 0.15% decursin (in DW); (3) the AGN group, which was treated with 2% AGN root extract (in DW). The AGN root extract has been used at about 2% solution in many studies [24,25]. The 2% AGN extract contained 0.15% decursin; therefore, the purchased decursin was used at 0.15%.

The hairs on the dorsal skin were shaved and 200 μL of the vehicle, decursin and/or AGN root extract, was applied on the skin twice a day for 17 days from 3 days after the shaving. The mice in each group were sacrificed at the designated times (1, 4, 7, 10, 14 and 17 days after the treatments of the vehicle, decursin, and AGN root extract).

### 2.5. Western Blot Analysis

To examine the expression levels of TNF-α, IL-1β, IL-4 and IL-13, western blot analyses were carried out in accordance to our previous study with modification [26]. Briefly, seven animals per group were sacrificed under deep anesthesia by intraperitoneal injection of pentobarbital sodium (60 mg/kg) (JW Pharm. Co., Ltd., Seoul, Republic of Korea). Thereafter, their dorsal skin tissues were removed and homogenized in 0.05 M phosphate-buffered saline (PBS, pH 7.4) containing 0.1 mM ethylene glycol-bis (2-aminoethyl ether)-N, N, N’, N’ tetraacetic acid (pH 8.0), 0.2% Nonidet P-40, 15 mM sodium pyrophosphate, 100 mM β-glycerophosphate, 0.01 M ethylenediaminetetraacetic acid (pH 8.0), 50 mM NaF, 150 mM NaCl, 2 mM sodium or thovanadate, 1 mM phenylmethylsulfonyl fluoride, and 1 mM dithiothreitol (DTT). Using 4–20% sodium dodecyl sulfate-polyacrylamide gel electrophoresis (SDS-PAGE), the tissues were separated for 3 h. Next, the resolved proteins were transferred to a nitrocellulose membrane for 2 h at 40 V. Each membrane was immunoreacted with respective primary antibody for 24 h at 4 °C: rabbit anti-TNF-α (diluted 1:1500, Abcam, Cambridge, UK), rabbit anti-IL-1β (diluted 1:1500, Abcam, Cambridge, UK), goat anti-IL-4 (diluted 1:1000, Santa Cruz Biotechnology, CA, USA), mouse anti-IL-13 (diluted 1:1000, Santa Cruz Biotechnology, CA, USA) and rabbit anti-β-actin (diluted 1:5000, Sigma–Aldrich, St. Louis, MO, USA). Subsequently, the individual immunoreacted membrane was exposed to a corresponding peroxidase-conjugated secondary antibody for 2 h at room temperature: goat anti-rabbit IgG (diluted 1:4000, Santa Cruz Biotechnology, CA, USA), goat anti-mouse IgG (diluted 1:5000, Merck KGaA, Darmstadt, Germany) and rabbit anti-goat IgG (diluted 1:5000, Merck KGaA, Darmstadt, Germany). Finally, the membrane blots were developed by using an enhanced luminol-based chemiluminescent (ECK) kit (Pierce Chemical, TX, USA).

### 2.6. Preparation of Tissue Sections

Tissue sections were prepared as follows, as previously described [26]. Briefly, ten animals per group were deeply anesthetized via intraperitoneal injection of pentobarbital sodium (60 mg/kg; JW Pharm. Co., Ltd., Seoul, Republic of Korea) at the designated times after the treatments with vehicle, AGN extract, and decursin. The whole bodies were rinsed with 0.1 M phosphate-buffered saline (PBS, pH 7.4) via the ascending aorta and fixed by perfusion with 4% paraformaldehyde (in 0.1 M phosphate buffer) solution. Subsequently, their dorsal skin tissues were harvested and post-fixed in the same fixative for 4 h at room temperature. Thereafter, the skin tissues were embedded in paraffin blocks according to the general method and sectioned into 8-μm thickness on a microtome (Leica, Wetzlar, Germany).

### 2.7. Hematoxylin and Eosin (H&E) Staining

To examine alterations in the epidermis and dermis, including hair follicles in each group, H and E staining was done according to a previous study [27]. Briefly, the prepared sections were stained with a hematoxylin solution and an eosin solution in consecutive order. The stained sections were dehydrated through serial ethanol. Finally, the stained sections were mounted with Canada balsam (Kanto Chemical, Tokyo, Japan) and covered with glass.

### 2.8. Immunohistochemistry

Immunohistochemical staining was done to compare changes in inflammatory cytokines between the groups. Briefly, the prepared sections were incubated in a 0.3% hydrogen peroxide (H_2_O_2_) solution and immersed in a 10% normal donkey serum (in 0.1M PBS, pH 7.4) solution for 30 min, respectively, at room temperature, as previously described [28]. Next, they were washed with PBS and immunoreacted with each primary antibody overnight at 4 °C. The primary antibodies were (1) and (2) rabbit anti-TNF-α (1:1000; Abcam, Cambridge, UK) and rabbit anti-IL-1β (1:200; Abcam, Cambridge, UK) for pro-inflammatory cytokines, (3) and (4) rabbit anti-IL-4 (1:250; Santa Cruz Biotechnology, CA, USA) and, mouse anti-IL-13 (1:250; Santa Cruz Biotechnology, CA, USA) for anti-inflammatory cytokines, and (5) rabbit anti-HMGB1 (1:1000; Abcam, Cambridge, UK) for inflammatory mediator. These immunoreacted sections were subsequently reacted with secondary antibody (biotinylated anti-rabbit IgG; 1:200; Vector, CA, USA) for 2 h at room temperature and immersed in an avidin-biotin complex (1:250; Vector, CA, USA) for 1 h at room temperature. Finally, these sections were colorized in brown with 0.05% 3, 3′-diaminobenzidine tetrahydrochloride (Sigma–Aldrich, St. Louis, MO, USA) solution (in 0.1 M PBS).

### 2.9. Data Analysis

Hair growth promoting scores were measured based on the hair criteria, which were described by a published study [29]. The criteria were as follows: 0, pink-colored shaved dorsal skin area; 1, gray-colored shaved dorsal skin area; 2, black-colored shaved dorsal skin; 3, initiation of hair growth on the shaved dorsal skin; 4, fully grown hair on the shaved dorsal skin.

Histopathological changes, including numbers of hair follicles, were analyzed according to the method by Her et al., (2019). Briefly, 7 sections stained with H and E in each mouse were obtained. Images of the sections were captured using a light microscope (AxioM1) (Carl Zeiss, Oberkochen, Germany) equipped with a DP72 digital camera (Olympus, Tokyo, Japan) connected to a PC monitor. Hair follicles were counted in 300 μm^2^ of the tissue area.

Immunoreactivities of TNF-α, interleukins and HMGB1 were analyzed from the 7 sections per mouse according to a published protocol [26] with modification. Their images were taken in the tissue area of 70 μm^2^ of the dorsal skin using an AxioM1 light microscope (Carl Zeiss, Oberkochen, Germany) equipped with a digital camera (DP72) (Olympus, Tokyo, Japan). The captured images were calibrated into an array of 512 × 512 pixels, and each relative optical density (ROD) was quantified by a gray-scaled system (range, 0–255) using an Image J software (version 1.46) (National Institutes of Health, Bethesda, MD, USA). The ratio of ROD was calibrated as a%. The ROD of the control group was considered to be 100%.

Consistent with a previous study [30], analyses of the Western blotting were performed. Briefly, using a ChemiDoc Imaging System (Bio–Rad Laboratories, Inc., Hercules, CA, USA), the bands of TNF-α, IL-1β, IL-4 and IL-13 were scanned, and densitometric analysis for the quantification of the bands was performed using Scion Image software (Scion Corp., Frederick, MD, USA). Each expression rate of the target protein was normalized through the corresponding expression rate of β-actin.

### 2.10. Statistical Analysis

Data obtained in the present study were shown as the mean ± standard error of mean (SEM). Using SPSS 18.0 software (SPSS, IL, USA), the present data were statistically analyzed. The statistical significance was established using Two-way analysis of variance (ANOVA) with a post hoc Tukey’s test for all pairwise multiple comparisons. The significant differences were designated when the P value was less than 0.05.

## 3. Results

### 3.1. Decursin of AGN Extract

**Figure 1:**
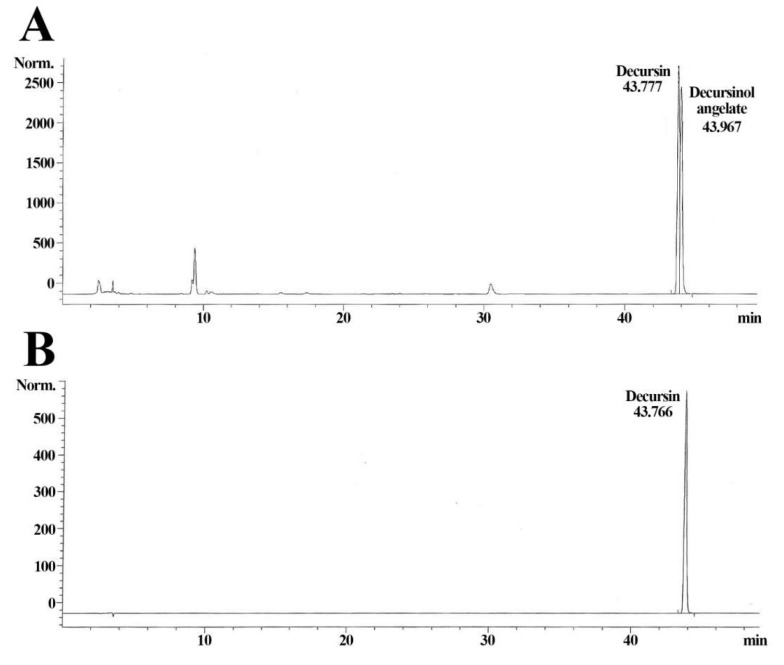
Representative HPLC chromatograms of AGN extract (**A**) and standard decursin (**B**). Retention times of the AGN extract are 43.777 min (decursin) and 43.967 (decursinol angelate) respectively, and the retention time of the standard decursin is 43.766 min.

Shown in Figure 1, decursin and decursinol angelate were detected (Figure 1A). The quantity of the decursin was calculated with each peak area, and the quantity of the decursin in the AGN extract was approximately 7.3 ± 2% (Figure 1A,B).

### 3.2. Hair Growth

**Figure 2:**
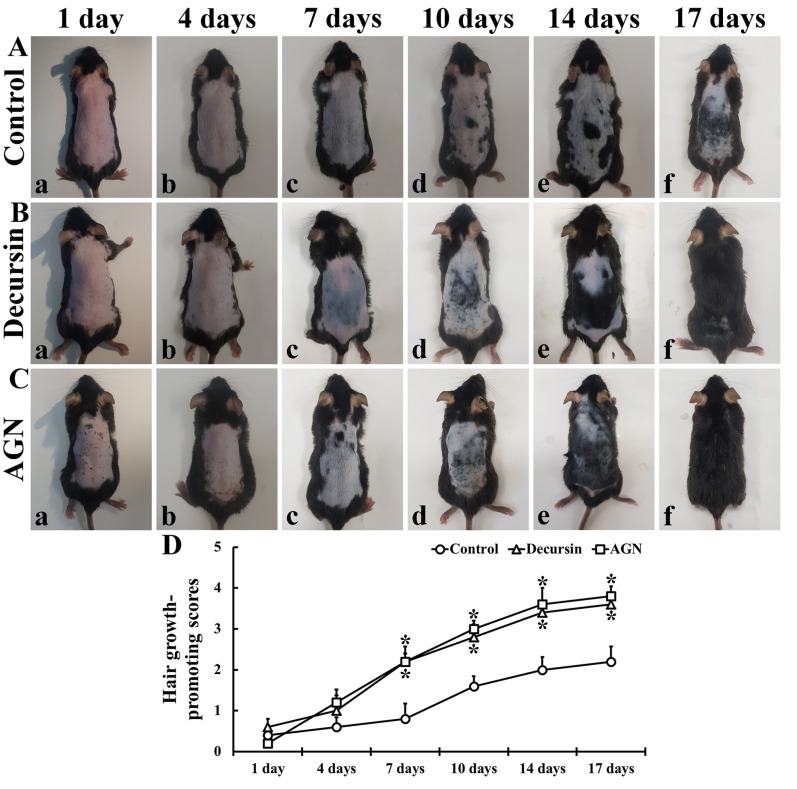
The processes of hair growth in the dorsal skin of the control (**A**), decursin (**B**), and AGN (**C**) groups. Seen in the control group, short hairs are shown at 10 days. However, in the decursin and AGN groups, hairs are shown at 7 days. Occurring by 17 days, complete hair growth is shown in the decursin and AGN groups. (**D**) Hair growth-promoting scores (*n* = 10 in each group; * *p* < 0.05 versus control group). The bars indicate the means ± SEM.

Seen in the control group, maintenance of pinkish dorsal skin was observed until seven days after the topical application of DW (Figure 2(Aa–Ac)). Regarding this group, grayish dorsal skin with short hairs was found from 10 days after the DW application and, thereafter, the hair growth progressed, showing that black skin was examined at 17 days after the DW application (Figure 2(Aa–Ac)).

Until four days after the application of decursin or AGN extract, the progress of hair growth was not significantly different from that in the control group (Figure 2(Ba,Bb,Ca,Cb,D)). However, the progress of hair growth was significantly facilitated from seven days after treatment with decursin and AGN extract compared to that in the control group (Figure 2(Bc–Bf,Cc–Cf,D)). Concerning these mice, completely grown hairs were found at 17 days after the treatment (Figure 2(Bf,Cf)).

### 3.3. Hair Follicles

**Figure 3:**
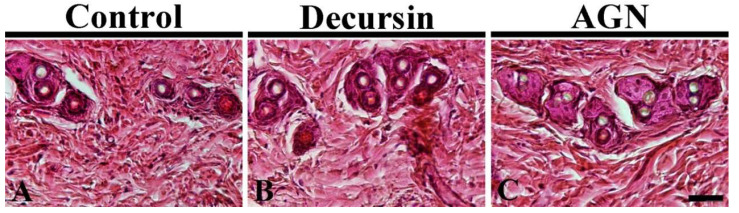
H and E staining in the dorsal skin of the control (**A**), decursin (**B**) and AGN (**C**) groups at 17 days after topical application of DW, decursin and AGN, respectively. There is no significant difference in the histology stained with H and E among the three groups (*n* = 10 in each group). Scale bar = 40 μm.

Found in the control group, at 17 days after DW treatment, hair follicles showed fine dyeability of H and E, and typical morphology of the hair follicles was observed (Figure 3A). Regarding the decursin and AGN groups, the stainability of H and E and the morphology of the hair follicles were not significantly different compared to those in the control group (Figure 3B,C).

### 3.4. Levels of Pro- and Anti-Inflammatory Cytokines

**Figure 4:**
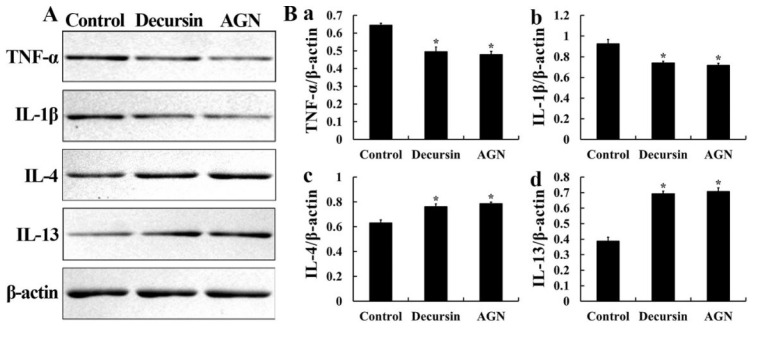
Representative blot images of TNF-α, IL-1β, IL-4 and IL-13 (**A**) in mouse dorsal skin and each protein expression normalized to β-actin (**Ba**–**d**) (*n* = 7 in each group; * *p* < 0.05 versus control group). The bars indicate the means ± SEM.

Regarding the decursin and AGN groups, protein levels of pro-inflammatory cytokines (TNF-α and IL-1β) in the dorsal skin tissue were significantly reduced (TNF-α, 76.9% and 74.3%, respectively, of the control group; IL-1β, 80.0% and 77.4%, respectively, of the control group) compared to those in the control group (Figure 4A or Figure 4(Ba,Bb)). Concerning the AGN group, particularly, both TNF-α and IL-1β levels were slightly lower than those in the decursin group (Figure 4(Ba,Bb)).

Conversely, the protein levels of anti-inflammatory cytokines (IL-4 and IL-13) were significantly increased (IL-4, 120.7% and 124.8%, respectively, of the control group; IL-13, 178.6% and 182.3%, respectively, of the control group) in the decursin and AGN groups compared to those in the control group (Figure 4A or Figure 4(Bc,Bd)). Particularly, in the AGN group, the levels of IL-4 and IL-13 were slightly higher than those in the decursin group (Figure 4(Bc,Bd)).

### 3.5. Pro-Inflammatory Cytokines

**Figure 5:**
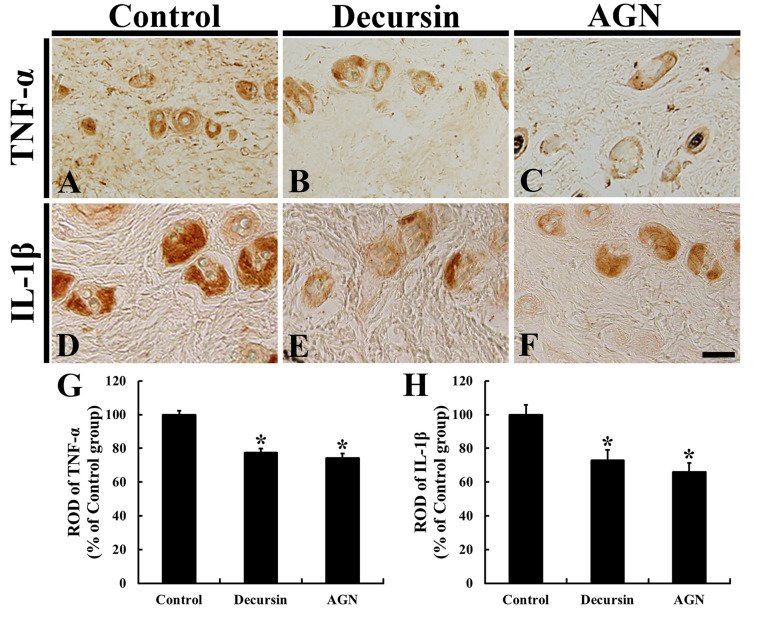
Immunohistochemistry for TNF-α (**A**–**C**) and IL-1β (**D**–**F**) in the dorsal skin of the control (**A**,**D**), decursin (**B**,**E**) and AGN (**C**,**F**) groups at 17 days. Regarding the decursin and AGN groups, both TNF-α and IL-1β immunoreactivities are significantly weakened compared to those in the control group. Scale bar = 40 μm. (**G**,**H**): ROD of TNF-α (**G**) and IL-1β (**H**) (*n* = 10 in each group; * *p* < 0.05 versus control group). The bars indicate the means ± SEM.

Immunoreactivity of TNF-α in the dorsal skin of the control group was shown in the hair follicles (Figure 5A). However, in the decursin and AGN groups, the TNF-α immunoreactivity was significantly weakened (about 77.5% and 74.1%, respectively, of the control group) compared to that in the control group (Figure 5B,C), showing that TNF-α immunoreactivity in the AGN group was slightly lower than that in the decursin group (Figure 5G).

IL-1β immunoreactivity in the dorsal skin of the control group was strong in the hair follicles (Figure 5D). Regarding the decursin and AGN groups, however, the IL-1β immunoreactivity was significantly reduced (about 72.9% and 66.0%, respectively, of the control group) compared to that in the control group (Figure 5E,F), showing that IL-1β immunoreactivity in the AGN group was lower than that in the decursin group (Figure 5H).

### 3.6. Anti-Inflammatory Cytokines

**Figure 6:**
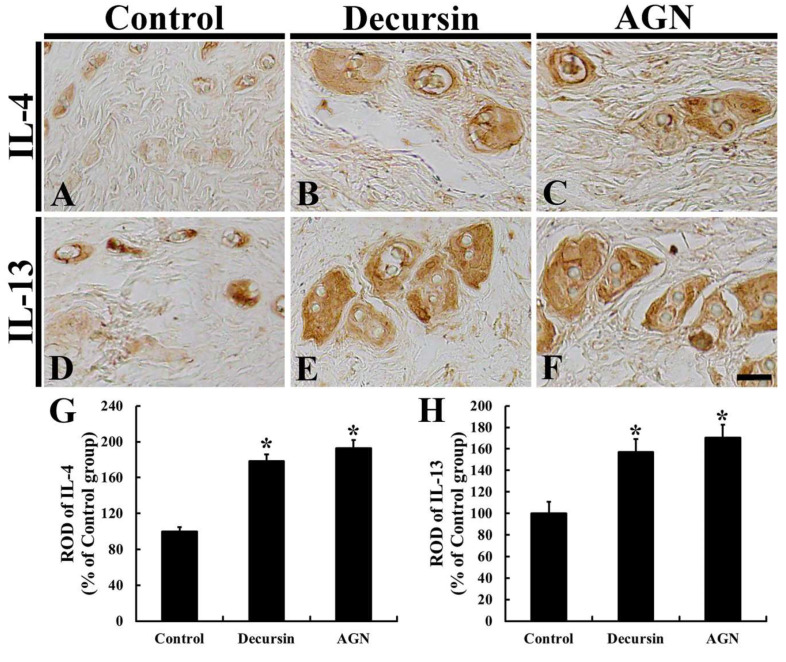
Immunohistochemistry for IL-4 (**A**–**C**) and IL-13 (**D**–**F**) in the dorsal skin of the control (**A**,**D**), decursin (**B**,**E**) and AGN (**C**,**F**) groups at 17 days. Regarding the decursin and AGN groups, significantly enhanced immunoreactivities of both IL-4 and IL-13 are found compared to those in the control group. Scale bar = 40 μm. (**G**,**H**) ROD of IL-4 (**G**) and IL-13 (**H**) (*n* = 10 in each group; * *p* < 0.05 versus control group). The bars indicate the means ± SEM.

Regarding the control group, IL-4 immunoreactivity in the dorsal skin was fundamentally observed in the hair follicles (Figure 6A). Concerning the decursin and AGN groups, contrastively, IL-4 immunoreactivity was significantly higher (about 178.1% and 193.0%, respectively, of the control group) than that in the control group (Figure 6B,C), showing that IL-4 immunoreactivity in the AGN group was slightly higher than that in the decursin group (Figure 6D).

Additionally, IL-13 immunoreactivity in the dorsal skin of the control group was shown in the hair follicles (Figure 6A). However, in the decursin and AGN groups, LI-13 immunoreactivity was significantly enhanced (about 157.0% and 170.4%, respectively, of the control group) compared to that in the control group (Figure 6B,C), showing that IL-13 immunoreactivity in the AGN group was higher than that in the decursin group (Figure 6D).

### 3.7. HMGB1 Immunoreactivity

**Figure 7:**
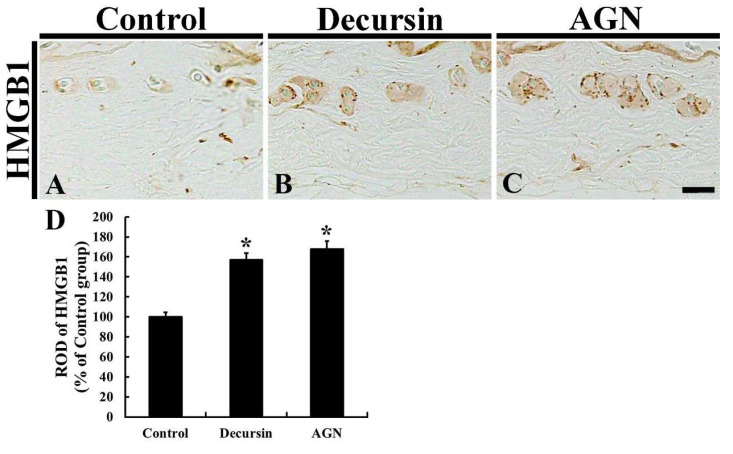
Immunohistochemistry for HMGB1 in the dorsal skin of the control (**A**), decursin (**B**) and AGN (**C**) groups at 17 days. In the decursin and AGN groups, significantly strengthened HMGB1 immunoreactivity is detected compared to that in the control group. Scale bar = 40 μm. (**D**) ROD of HMGB1 (*n* = 10 in each group; * *p* < 0.05 versus control group). The bars indicate the means ± SEM.

Regarding the control group, the immunoreactivity of HMGB1, an inflammatory mediator, was essentially detected in the hair follicles in the dorsal skin (Figure 7A). Concerning the decursin and AGN groups, HMGB1 immunoreactivity was significantly enhanced (157.0% and 167.6%, respectively, of the control group) compared to that in the control group (Figure 7B,C), showing that HMGB1 immunoreactivity in the AGN group was higher than that in the decursin group.

## 4. Discussion

Since a large number of herbal medicines and their secondary metabolites display anti-inflammatory effects [12,13,28,31], they have been traditionally used as remedies and currently utilized as complementary medicines [31,32]. Among medicinal plants, the AGN extract was verified to have excellent anti-inflammatory effects. The ethanol extract of AGN, for instance, displayed anti-inflammatory effects by reducing the production of histamine and atopy-related cytokines and suppressing the levels of COX-2, NF-κB, and Iκ-Bα in both in vivo and in vitro models of atopic dermatitis [12]. Additionally, AGN extract prevented epithelial injury in colonic tissues of a murine model of dextran sulfate sodium-induced ulcerative colitis by moderating the activation of inflammatory mediators, such as IL-6, TNF-α, prostaglandin E2 (PGE2), and cyclooxygenase-2 (COX-2) [33]. Moreover, Shin et al., (2009) demonstrated that the ethanol extract of AGN ameliorated inflammatory responses by blocking the TNF-α-nitric oxide (NO) pathway and attenuating the infiltration of immunocytes in a mouse model of carrageenan-air pouch inflammation [34].

Some precedent studies reported the composition of AGN root extract, as shown in Table 1. Among the components, coumarin derivatives such as decursin, decursinol, and decursinol angelate comprise a majority of the composition. Seo et al. (2009) reported that decursinol exerted a pain-killing effect, which was related to an acetaminophen-induced analgesic mechanism [35]. Additionally, decursinol angelate, a structural isomer of the decursin, showed inhibitory actions against the infiltration of immunocytes, oxidative stress, and the production of pro-inflammatory cytokines in mouse ear skin inflammation induced by the topical application of tetradecanoyl phorbol-13-acetate [36]. Particularly, decursin is abundantly contained in AGN root extract and exerts a wide range of bioactive activities [37,38]. A study recently reported that decursin displayed anti-inflammatory activity via down-regulation of the TLR4/JNK signaling pathway provoked by PRP4 expression in lipopolysaccharide (LPS)-induced skin cancer (B16–F10) cells [22]. Furthermore, decursin inhibited the polarization of macrophages by regulating the NF-κB/MAPK signaling pathway in LPS-stimulated Raw 264.7 cells [39]. Based on the anti-inflammatory effects of decursin described above, this study conducted an HPLC analysis of decursin and verified that AGN root extract contained 7.3 ± 2% decursin. The anti-inflammatory and hair growth effects of the extract also were examined and compared to those of commercially purchased decursin in mouse dorsal skin.

Here, the protein levels and immunoreactivity of TNF-α and IL-1β were significantly decreased, whereas the IL-4 and IL-13 levels and immunoreactivity were significantly increased compared to those in the control group. Taken together, the treatment of mouse dorsal skin with AGN root extract containing decursin in mouse dorsal skin can promote hair growth via regulation of pro- and/or anti-inflammatory cytokines. We, therefore, suggest that AGN root extract as well as decursin can be utilized as materials for developing hair growth facilitators.

During this study, 2% AGN root extract was applied to the dorsal skin according to some precedent studies that investigated the hair growth potential of extracts from natural resources [24,25]. Seen in our current study, completely grown hairs in the dorsal skin were found 17 days after treatment with AGN root extract or decursin. Regarding the control group, the hairs were not fully grown 17 days after treatment with DW. Additionally, treatment with AGN root extract and decursin did not lead to morphological changes in the hair follicles stained with H and E.

The results suggest that a leading contribution to the hair growth-promoting effect of AGN extract and decursin was the modulation of both pro- and anti-inflammatory cytokines. The Western blot and immunohistochemical analyses of TNF-α, IL-1β, IL-4, and IL-13 in the dorsal skin showed that AGN extract and decursin significantly reduced pro-inflammatory cytokines (TNF-α and IL-1β), which trigger progressive inflammatory responses [6,8]. Conversely, they increased anti-inflammatory cytokine (IL-4 and IL-13) levels which can inhibit the synthesis of pro-inflammatory cytokines and act as antagonists of pro-inflammatory cytokine receptors [6,7]. Interestingly, treatment with AGN root extract showed a little more facilitation of hair growth than that by decursin, indicating that the regulation of pro- and anti-inflammatory cytokines was slightly more effective compared to that of the decursin group. These findings might have been affected by components of the AGN extract, such as decursinol angelate and nodakenin. Decursinol angelate possesses anti-inflammatory activity [36]. Furthermore, treatment with nodakenin contained in AGN extract was shown to ameliorate skin lesions in 2, 4-dinitrochlorobenzene (DNCB)-induced atopic dermatitis in mice via suppressing IgE-mediated allergic responses [42].

It has been widely accepted that inflammatory responses are associated with hair loss [9,43,44]. The development of hair loss is closely associated with activated immunocytes and migration of the immunocytes to intra- and peri-follicular regions [9]. Additionally, it has been reported that interferon-gamma (an inflammatory cytokine) secreted by activated T cells is predominantly found in skin areas where hair loss is in progress [2]. Inflammation in the hair follicles was directly related to folliculitis decalvans [45]. Furthermore, it has been suggested that the modulation the inflammatory responses in the hair follicles is a key strategy for facilitating hair growth. Enhanced high-mobility group box 1 (HMGB1), an omnipresent nuclear protein, secreted by cellular nuclei after tissue damage acted as an inflammatory mediator and promoted hair growth through regulating prostaglandin metabolism in hair follicles [46], for example. Consistent with that report, our current study showed that HMGB1 immunoreactivity was enhanced in the follicles and/or peri-follicular regions of dorsal skin following the topical application of decursin and AGN root extract.

To summarize, this study showed that the topical application of 0.15% decursin or 2% AGN root extract facilitated hair growth in mouse dorsal skin. Additionally, such an effect might have resulted from the regulation of both pro- and anti-inflammatory cytokines by AGN extract and decursin. Based on these results, we suggest that decursin and AGN root extract can be utilized as materials to facilitate hair growth.

## Figures and Tables

**Table 1:** Composition of ethanol extract of AGN root.

Type	Components	
**Coumarin Derivatives**	Decursin	Major components
	Decursinol	
	Decursinol angelate	
	Nodakenin, 4′′-Hydroxytigloyldecursinol, 4′′-hydroxydecursin, (2′′S,3′′S)-epoxyangeloyldecursinol, (2′′R,3′′R)-epoxyangeloyldecursinol, marmesinin, columbianetin-O-β-D-glucopyranoside, etc.	Other components
**Saccharides**	Angelan (peptic polysaccharide)	Polysaccharide
	Arabinose, Galactose, Galacturonic acid (sugar acid), etc.	Monosaccharides
**Polyacetylene**	Octadeca-1,9-dien-4,6-diyn-3,8,18-triol, 18-acetoxy-octadeca-1,9-dien-4,6-diyn-3,8-diol, etc.	

The components are summarized from papers [38,40,41].
